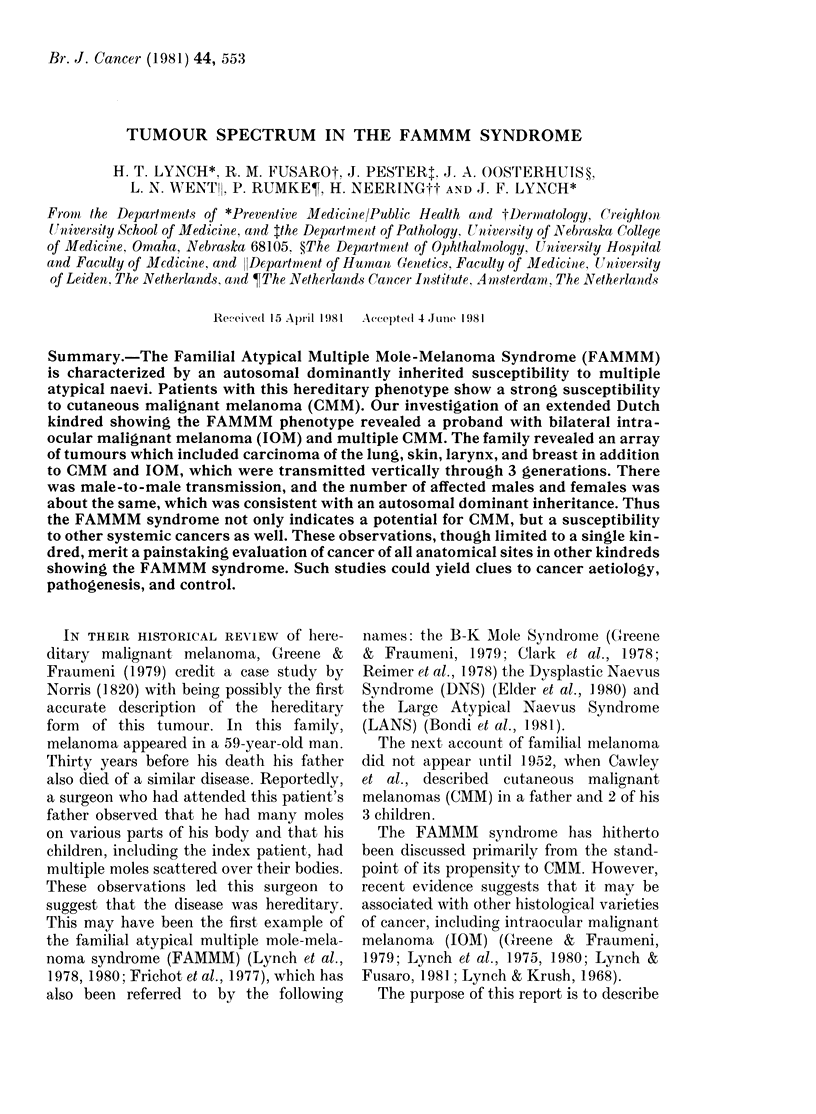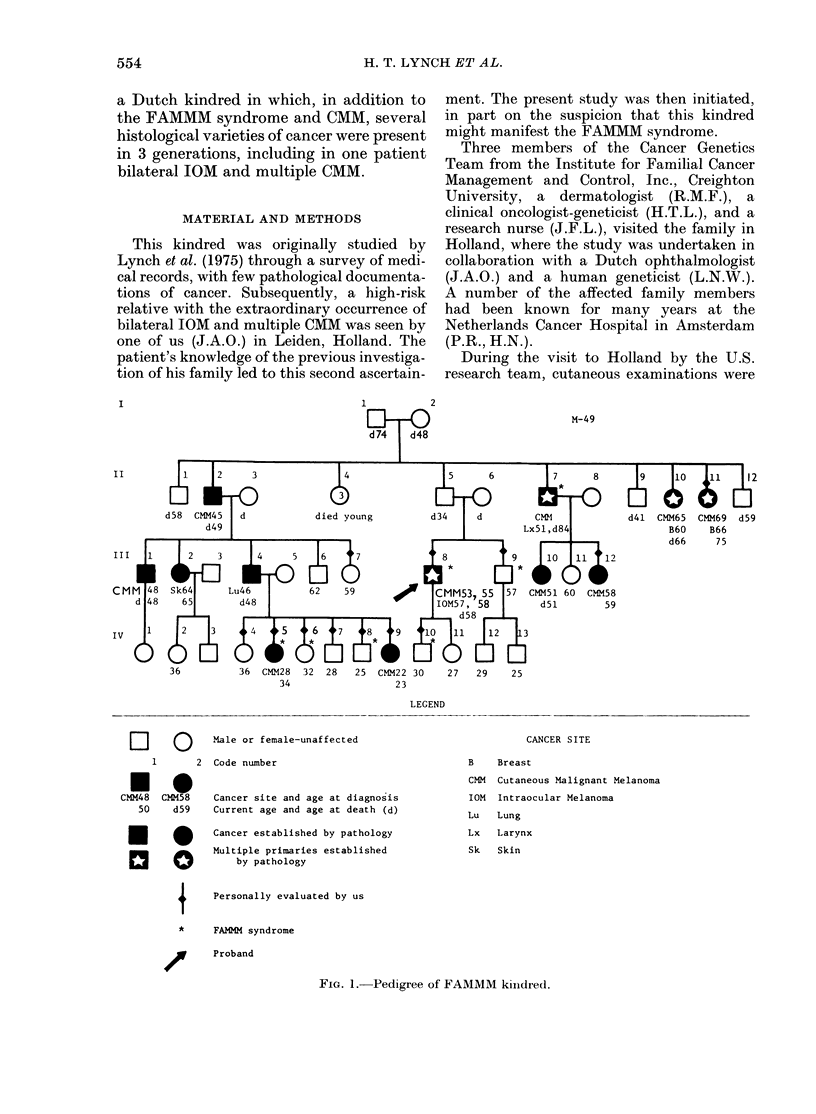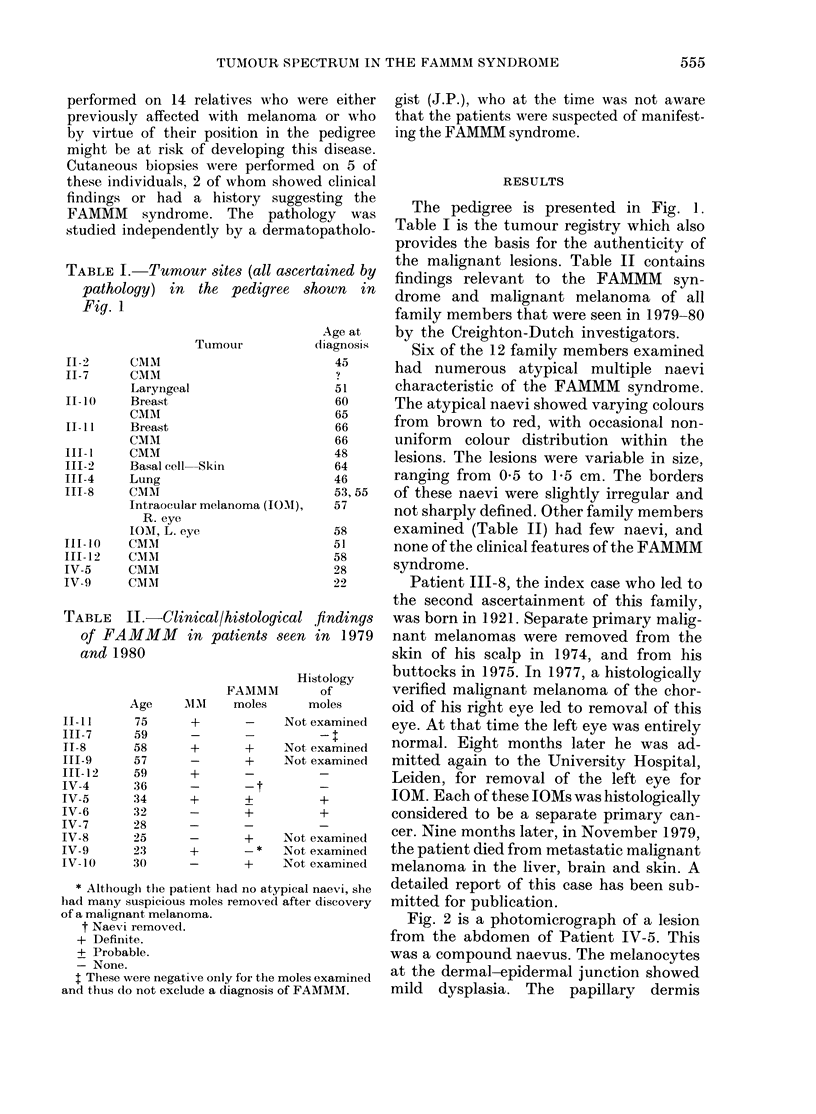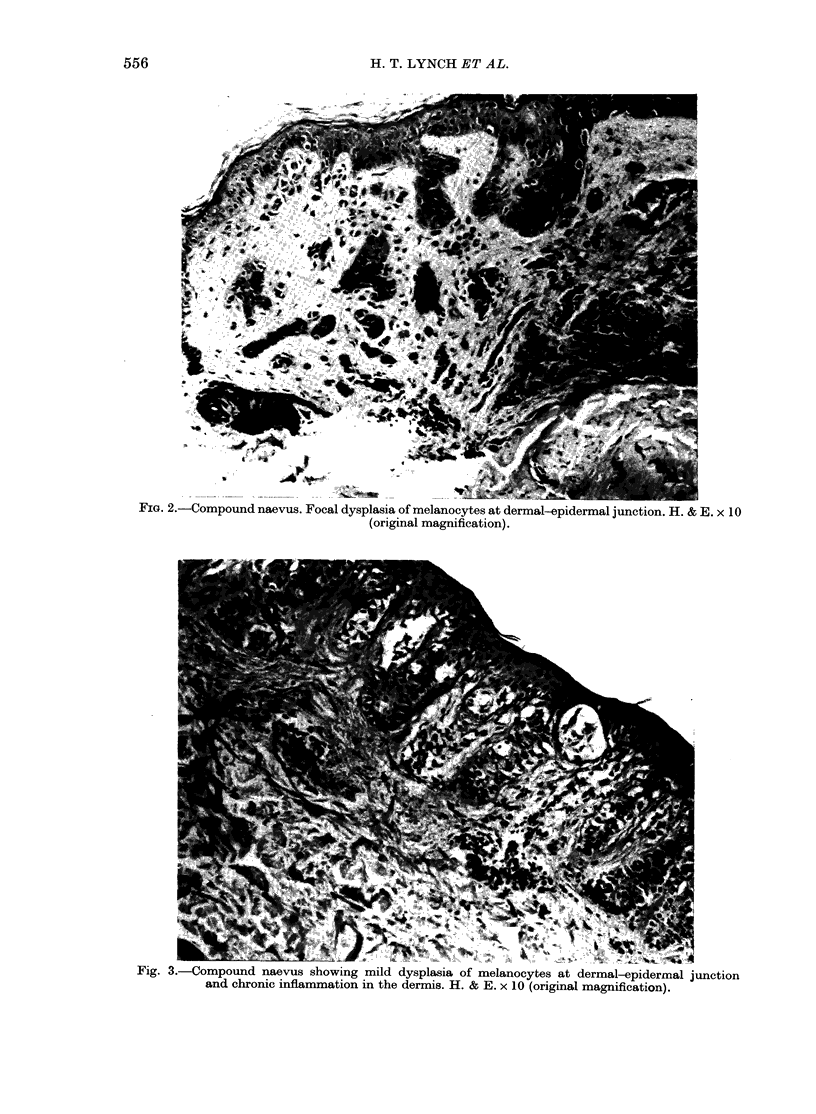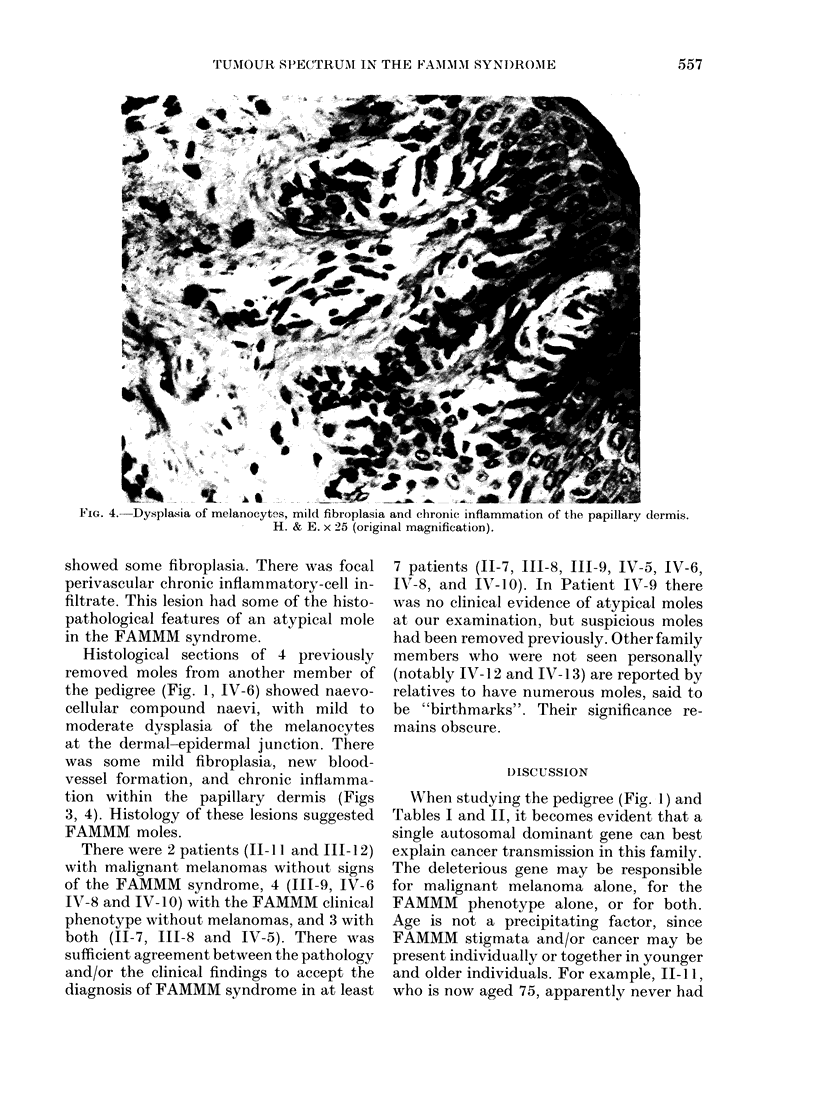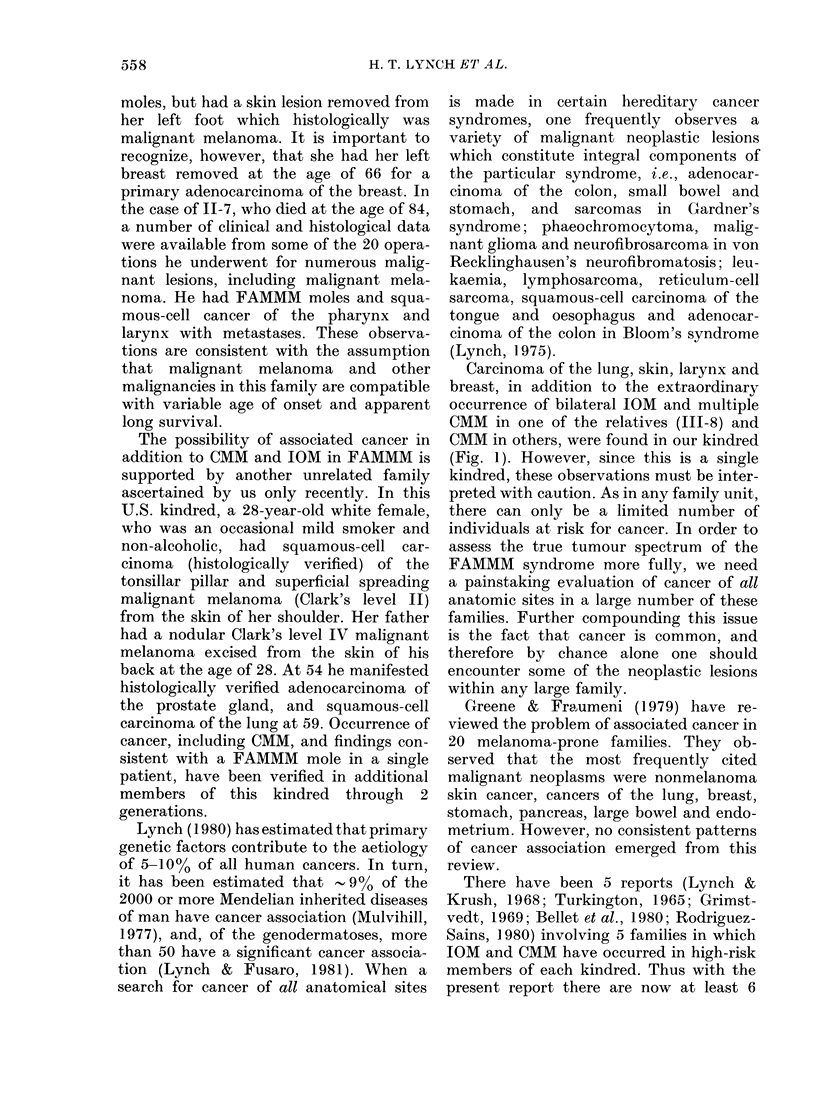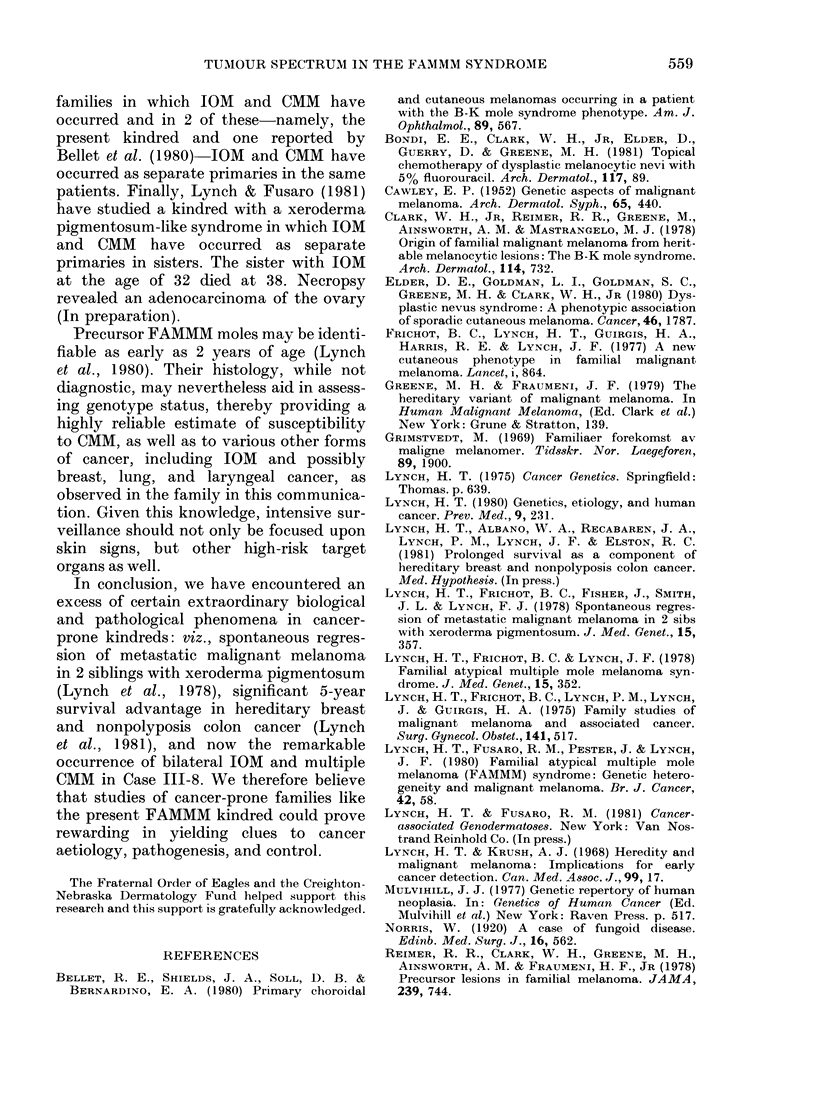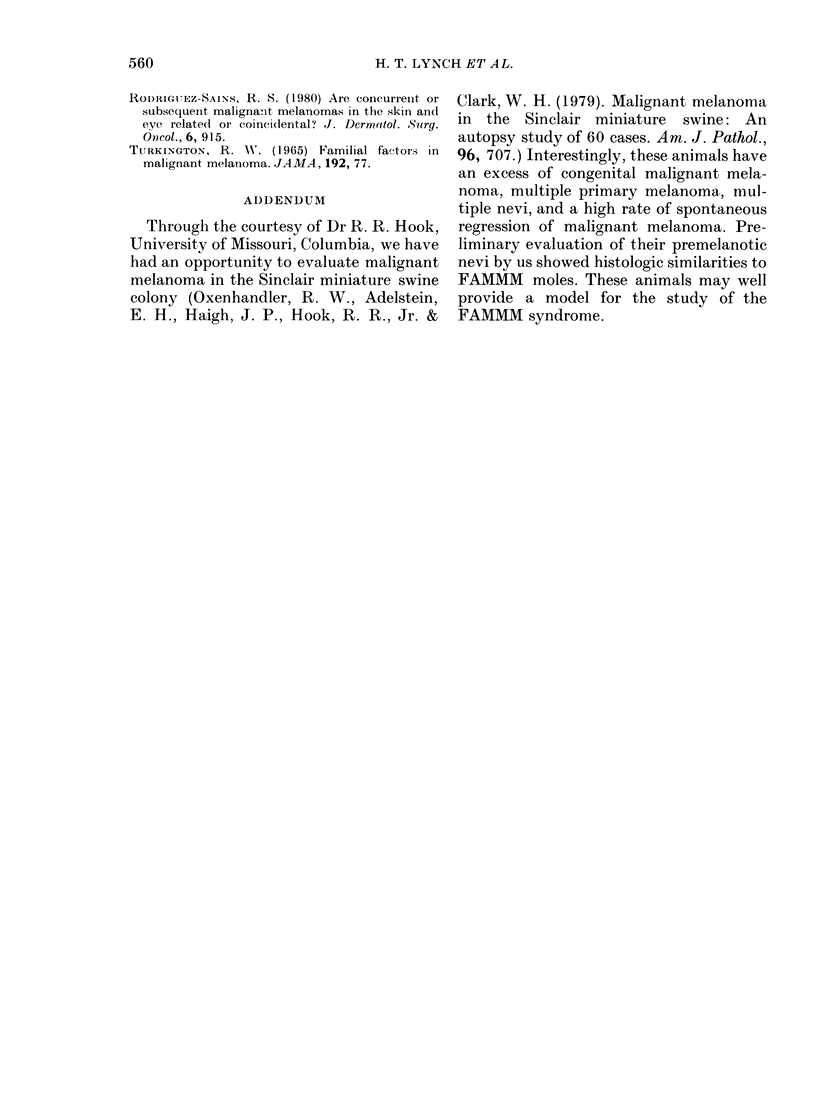# Tumour spectrum in the FAMMM syndrome.

**DOI:** 10.1038/bjc.1981.225

**Published:** 1981-10

**Authors:** H. T. Lynch, R. M. Fusaro, J. Pester, J. A. Oosterhuis, L. N. Went, P. Rumke, H. Neering, J. F. Lynch

## Abstract

**Images:**


*Br. J. Cancer* (1981) **44**, 553

### TUMOUR SPECTRUM IN THE FAMMM SYNDROME

H. T. LYNCH*, R. M. FUSARO†, J. PESTER‡, J. A. OOSTERHUIS§, L. N. WENT||, P. RUMKE¶, H. NEERING†† AND J. F. LYNCH*

*From the Departments of *Preventive Medicine/Public Health and †Dermatology, Creighton University School of Medicine, and ‡the Department of Pathology, University of Nebraska College of Medicine, Omaha, Nebraska 68105, §The Department of Ophthalmology, University Hospital and Faculty of Medicine, and ||Department of Human Genetics, Faculty of Medicine, University of Leiden, The Netherlands, and ¶The Netherlands Cancer Institute, Amsterdam, The Netherlands*

Received 15 April 1981 Accepted 4 June 1981

**Summary.—The Familial Atypical Multiple Mole-Melanoma Syndrome (FAMMM) is characterized by an autosomal dominantly inherited susceptibility to multiple atypical naevi. Patients with this hereditary phenotype show a strong susceptibility to cutaneous malignant melanoma (CMM). Our investigation of an extended Dutch kindred showing the FAMMM phenotype revealed a proband with bilateral intraocular malignant melanoma (IOM) and multiple CMM. The family revealed an array of tumours which included carcinoma of the lung, skin, larynx, and breast in addition to CMM and IOM, which were transmitted vertically through 3 generations. There was male-to-male transmission, and the number of affected males and females was about the same, which was consistent with an autosomal dominant inheritance. Thus the FAMMM syndrome not only indicates a potential for CMM, but a susceptibility to other systemic cancers as well. These observations, though limited to a single kindred, merit a painstaking evaluation of cancer of all anatomical sites in other kindreds showing the FAMMM syndrome. Such studies could yield clues to cancer aetiology, pathogenesis, and control.**

IN THEIR HISTORICAL REVIEW of hereditary malignant melanoma, Greene & Fraumeni (1979) credit a case study by Norris (1820) with being possibly the first accurate description of the hereditary form of this tumour. In this family, melanoma appeared in a 59-year-old man. Thirty years before his death his father also died of a similar disease. Reportedly, a surgeon who had attended this patient's father observed that he had many moles on various parts of his body and that his children, including the index patient, had multiple moles scattered over their bodies. These observations led this surgeon to suggest that the disease was hereditary. This may have been the first example of the familial atypical multiple mole-melanoma syndrome (FAMMM) (Lynch *et al.*, 1978, 1980; Frichot *et al.*, 1977), which has also been referred to by the following names: the B-K Mole Syndrome (Greene & Fraumeni, 1979; Clark *et al.*, 1978; Reimer *et al.*, 1978) the Dysplastic Naevus Syndrome (DNS) (Elder *et al.*, 1980) and the Large Atypical Naevus Syndrome (LANS) (Bondi *et al.*, 1981).

The next account of familial melanoma did not appear until 1952, when Cawley *et al.*, described cutaneous malignant melanomas (CMM) in a father and 2 of his 3 children.

The FAMMM syndrome has hitherto been discussed primarily from the standpoint of its propensity to CMM. However, recent evidence suggests that it may be associated with other histological varieties of cancer, including intraocular malignant melanoma (IOM) (Greene & Fraumeni, 1979; Lynch *et al.*, 1975, 1980; Lynch & Fusaro, 1981; Lynch & Krush, 1968).

The purpose of this report is to describe a Dutch kindred in which, in addition to the FAMMM syndrome and CMM, several histological varieties of cancer were present in 3 generations, including in one patient bilateral IOM and multiple CMM.

### MATERIAL AND METHODS

This kindred was originally studied by Lynch *et al.* (1975) through a survey of medical records, with few pathological documentations of cancer. Subsequently, a high-risk relative with the extraordinary occurrence of bilateral IOM and multiple CMM was seen by one of us (J.A.O.) in Leiden, Holland. The patient's knowledge of the previous investigation of his family led to this second ascertainment. The present study was then initiated, in part on the suspicion that this kindred might manifest the FAMMM syndrome.

Three members of the Cancer Genetics Team from the Institute for Familial Cancer Management and Control, Inc., Creighton University, a dermatologist (R.M.F.), a clinical oncologist-geneticist (H.T.L.), and a research nurse (J.F.L.), visited the family in Holland, where the study was undertaken in collaboration with a Dutch ophthalmologist (J.A.O.) and a human geneticist (L.N.W.). A number of the affected family members had been known for many years at the Netherlands Cancer Hospital in Amsterdam (P.R., H.N.).

During the visit to Holland by the U.S. research team, cutaneous examinations were

FIG. 1.—Pedigree of FAMMM kindred.

performed on 14 relatives who were either previously affected with melanoma or who by virtue of their position in the pedigree might be at risk of developing this disease. Cutaneous biopsies were performed on 5 of these individuals, 2 of whom showed clinical findings or had a history suggesting the FAMMM syndrome. The pathology was studied independently by a dermatopathologist (J.P.), who at the time was not aware that the patients were suspected of manifesting the FAMMM syndrome.

TABLE I.—*Tumour sites (all ascertained by pathology) in the pedigree shown in Fig.* 1

| | Tumour | Age at diagnosis |
|---|---|---|
| II-2 | CMM | 45 |
| II-7 | CMM | ? |
| | Laryngeal | 51 |
| II-10 | Breast | 60 |
| | CMM | 65 |
| II-11 | Breast | 66 |
| | CMM | 66 |
| III-1 | CMM | 48 |
| III-2 | Basal cell—Skin | 64 |
| III-4 | Lung | 46 |
| III-8 | CMM | 53, 55 |
| | Intraocular melanoma (IOM), R. eye | 57 |
| | IOM, L. eye | 58 |
| III-10 | CMM | 51 |
| III-12 | CMM | 58 |
| IV-5 | CMM | 28 |
| IV-9 | CMM | 22 |

TABLE II.—*Clinical/histological findings of FAMMM in patients seen in* 1979 *and* 1980

| | Age | MM | FAMMM moles | Histology of moles |
|---|---|---|---|---|
| II-11 | 75 | + | − | Not examined |
| III-7 | 59 | − | − | −‡ |
| II-8 | 58 | + | + | Not examined |
| III-9 | 57 | − | + | Not examined |
| III-12 | 59 | + | − | − |
| IV-4 | 36 | − | −† | − |
| IV-5 | 34 | + | ± | + |
| IV-6 | 32 | − | + | + |
| IV-7 | 28 | − | − | − |
| IV-8 | 25 | − | + | Not examined |
| IV-9 | 23 | + | −* | Not examined |
| IV-10 | 30 | − | + | Not examined |

* Although the patient had no atypical naevi, she had many suspicious moles removed after discovery of a malignant melanoma.

† Naevi removed.

+ Definite.

± Probable.

− None.

‡ These were negative only for the moles examined and thus do not exclude a diagnosis of FAMMM.

### RESULTS

The pedigree is presented in Fig. 1. Table I is the tumour registry which also provides the basis for the authenticity of the malignant lesions. Table II contains findings relevant to the FAMMM syndrome and malignant melanoma of all family members that were seen in 1979–80 by the Creighton-Dutch investigators.

Six of the 12 family members examined had numerous atypical multiple naevi characteristic of the FAMMM syndrome. The atypical naevi showed varying colours from brown to red, with occasional non-uniform colour distribution within the lesions. The lesions were variable in size, ranging from 0·5 to 1·5 cm. The borders of these naevi were slightly irregular and not sharply defined. Other family members examined (Table II) had few naevi, and none of the clinical features of the FAMMM syndrome.

Patient III-8, the index case who led to the second ascertainment of this family, was born in 1921. Separate primary malignant melanomas were removed from the skin of his scalp in 1974, and from his buttocks in 1975. In 1977, a histologically verified malignant melanoma of the choroid of his right eye led to removal of this eye. At that time the left eye was entirely normal. Eight months later he was admitted again to the University Hospital, Leiden, for removal of the left eye for IOM. Each of these IOMs was histologically considered to be a separate primary cancer. Nine months later, in November 1979, the patient died from metastatic malignant melanoma in the liver, brain and skin. A detailed report of this case has been submitted for publication.

Fig. 2 is a photomicrograph of a lesion from the abdomen of Patient IV-5. This was a compound naevus. The melanocytes at the dermal–epidermal junction showed mild dysplasia. The papillary dermis

FIG. 2.—Compound naevus. Focal dysplasia of melanocytes at dermal–epidermal junction. H. & E. × 10 (original magnification).

Fig. 3.—Compound naevus showing mild dysplasia of melanocytes at dermal–epidermal junction and chronic inflammation in the dermis. H. & E. × 10 (original magnification).

FIG. 4.—Dysplasia of melanocytes, mild fibroplasia and chronic inflammation of the papillary dermis. H. & E. x 25 (original magnification).

showed some fibroplasia. There was focal perivascular chronic inflammatory-cell infiltrate. This lesion had some of the histopathological features of an atypical mole in the FAMMM syndrome.

Histological sections of 4 previously removed moles from another member of the pedigree (Fig. 1, IV-6) showed naevocellular compound naevi, with mild to moderate dysplasia of the melanocytes at the dermal–epidermal junction. There was some mild fibroplasia, new blood-vessel formation, and chronic inflammation within the papillary dermis (Figs 3, 4). Histology of these lesions suggested FAMMM moles.

There were 2 patients (II-11 and III-12) with malignant melanomas without signs of the FAMMM syndrome, 4 (III-9, IV-6 IV-8 and IV-10) with the FAMMM clinical phenotype without melanomas, and 3 with both (II-7, III-8 and IV-5). There was sufficient agreement between the pathology and/or the clinical findings to accept the diagnosis of FAMMM syndrome in at least 7 patients (II-7, III-8, III-9, IV-5, IV-6, IV-8, and IV-10). In Patient IV-9 there was no clinical evidence of atypical moles at our examination, but suspicious moles had been removed previously. Other family members who were not seen personally (notably IV-12 and IV-13) are reported by relatives to have numerous moles, said to be "birthmarks". Their significance remains obscure.

### DISCUSSION

When studying the pedigree (Fig. 1) and Tables I and II, it becomes evident that a single autosomal dominant gene can best explain cancer transmission in this family. The deleterious gene may be responsible for malignant melanoma alone, for the FAMMM phenotype alone, or for both. Age is not a precipitating factor, since FAMMM stigmata and/or cancer may be present individually or together in younger and older individuals. For example, II-11, who is now aged 75, apparently never had moles, but had a skin lesion removed from her left foot which histologically was malignant melanoma. It is important to recognize, however, that she had her left breast removed at the age of 66 for a primary adenocarcinoma of the breast. In the case of II-7, who died at the age of 84, a number of clinical and histological data were available from some of the 20 operations he underwent for numerous malignant lesions, including malignant melanoma. He had FAMMM moles and squamous-cell cancer of the pharynx and larynx with metastases. These observations are consistent with the assumption that malignant melanoma and other malignancies in this family are compatible with variable age of onset and apparent long survival.

The possibility of associated cancer in addition to CMM and IOM in FAMMM is supported by another unrelated family ascertained by us only recently. In this U.S. kindred, a 28-year-old white female, who was an occasional mild smoker and non-alcoholic, had squamous-cell carcinoma (histologically verified) of the tonsillar pillar and superficial spreading malignant melanoma (Clark's level II) from the skin of her shoulder. Her father had a nodular Clark's level IV malignant melanoma excised from the skin of his back at the age of 28. At 54 he manifested histologically verified adenocarcinoma of the prostate gland, and squamous-cell carcinoma of the lung at 59. Occurrence of cancer, including CMM, and findings consistent with a FAMMM mole in a single patient, have been verified in additional members of this kindred through 2 generations.

Lynch (1980) has estimated that primary genetic factors contribute to the aetiology of 5–10% of all human cancers. In turn, it has been estimated that ~9% of the 2000 or more Mendelian inherited diseases of man have cancer association (Mulvihill, 1977), and, of the genodermatoses, more than 50 have a significant cancer association (Lynch & Fusaro, 1981). When a search for cancer of *all* anatomical sites is made in certain hereditary cancer syndromes, one frequently observes a variety of malignant neoplastic lesions which constitute integral components of the particular syndrome, *i.e.*, adenocarcinoma of the colon, small bowel and stomach, and sarcomas in Gardner's syndrome; phaeochromocytoma, malignant glioma and neurofibrosarcoma in von Recklinghausen's neurofibromatosis; leukaemia, lymphosarcoma, reticulum-cell sarcoma, squamous-cell carcinoma of the tongue and oesophagus and adenocarcinoma of the colon in Bloom's syndrome (Lynch, 1975).

Carcinoma of the lung, skin, larynx and breast, in addition to the extraordinary occurrence of bilateral IOM and multiple CMM in one of the relatives (III-8) and CMM in others, were found in our kindred (Fig. 1). However, since this is a single kindred, these observations must be interpreted with caution. As in any family unit, there can only be a limited number of individuals at risk for cancer. In order to assess the true tumour spectrum of the FAMMM syndrome more fully, we need a painstaking evaluation of cancer of *all* anatomic sites in a large number of these families. Further compounding this issue is the fact that cancer is common, and therefore by chance alone one should encounter some of the neoplastic lesions within any large family.

Greene & Fraumeni (1979) have reviewed the problem of associated cancer in 20 melanoma-prone families. They observed that the most frequently cited malignant neoplasms were nonmelanoma skin cancer, cancers of the lung, breast, stomach, pancreas, large bowel and endometrium. However, no consistent patterns of cancer association emerged from this review.

There have been 5 reports (Lynch & Krush, 1968; Turkington, 1965; Grimstvedt, 1969; Bellet *et al.*, 1980; Rodriguez-Sains, 1980) involving 5 families in which IOM and CMM have occurred in high-risk members of each kindred. Thus with the present report there are now at least 6

families in which IOM and CMM have occurred and in 2 of these—namely, the present kindred and one reported by Bellet *et al.* (1980)—IOM and CMM have occurred as separate primaries in the same patients. Finally, Lynch & Fusaro (1981) have studied a kindred with a xeroderma pigmentosum-like syndrome in which IOM and CMM have occurred as separate primaries in sisters. The sister with IOM at the age of 32 died at 38. Necropsy revealed an adenocarcinoma of the ovary (In preparation).

Precursor FAMMM moles may be identifiable as early as 2 years of age (Lynch *et al.*, 1980). Their histology, while not diagnostic, may nevertheless aid in assessing genotype status, thereby providing a highly reliable estimate of susceptibility to CMM, as well as to various other forms of cancer, including IOM and possibly breast, lung, and laryngeal cancer, as observed in the family in this communication. Given this knowledge, intensive surveillance should not only be focused upon skin signs, but other high-risk target organs as well.

In conclusion, we have encountered an excess of certain extraordinary biological and pathological phenomena in cancer-prone kindreds: *viz.*, spontaneous regression of metastatic malignant melanoma in 2 siblings with xeroderma pigmentosum (Lynch *et al.*, 1978), significant 5-year survival advantage in hereditary breast and nonpolyposis colon cancer (Lynch *et al.*, 1981), and now the remarkable occurrence of bilateral IOM and multiple CMM in Case III-8. We therefore believe that studies of cancer-prone families like the present FAMMM kindred could prove rewarding in yielding clues to cancer aetiology, pathogenesis, and control.

The Fraternal Order of Eagles and the Creighton-Nebraska Dermatology Fund helped support this research and this support is gratefully acknowledged.

### REFERENCES

Bellet, R. E., Shields, J. A., Soll, D. B. & Bernardino, E. A. (1980) Primary choroidal and cutaneous melanomas occurring in a patient with the B-K mole syndrome phenotype. *Am. J. Ophthalmol.*, **89**, 567.

Bondi, E. E., Clark, W. H., Jr, Elder, D., Guerry, D. & Greene, M. H. (1981) Topical chemotherapy of dysplastic melanocytic nevi with 5% fluorouracil. *Arch. Dermatol.*, **117**, 89.

Cawley, E. P. (1952) Genetic aspects of malignant melanoma. *Arch. Dermatol. Syph.*, **65**, 440.

Clark, W. H., Jr, Reimer, R. R., Greene, M., Ainsworth, A. M. & Mastrangelo, M. J. (1978) Origin of familial malignant melanoma from heritable melanocytic lesions: The B-K mole syndrome. *Arch. Dermatol.*, **114**, 732.

Elder, D. E., Goldman, L. I., Goldman, S. C., Greene, M. H. & Clark, W. H., Jr (1980) Dysplastic nevus syndrome: A phenotypic association of sporadic cutaneous melanoma. *Cancer*, **46**, 1787.

Frichot, B. C., Lynch, H. T., Guirgis, H. A., Harris, R. E. & Lynch, J. F. (1977) A new cutaneous phenotype in familial malignant melanoma. *Lancet*, i, 864.

Greene, M. H. & Fraumeni, J. F. (1979) The hereditary variant of malignant melanoma. In *Human Malignant Melanoma*, (Ed. Clark *et al.*) New York: Grune & Stratton, 139.

Grimstvedt, M. (1969) Familiaer forekomst av maligne melanomer. *Tidsskr. Nor. Laegeforen*, **89**, 1900.

Lynch, H. T. (1975) *Cancer Genetics*. Springfield: Thomas. p. 639.

Lynch, H. T. (1980) Genetics, etiology, and human cancer. *Prev. Med.*, **9**, 231.

Lynch, H. T., Albano, W. A., Recabaren, J. A., Lynch, P. M., Lynch, J. F. & Elston, R. C. (1981) Prolonged survival as a component of hereditary breast and nonpolyposis colon cancer. *Med. Hypothesis.* (In press.)

Lynch, H. T., Frichot, B. C., Fisher, J., Smith, J. L. & Lynch, F. J. (1978) Spontaneous regression of metastatic malignant melanoma in 2 sibs with xeroderma pigmentosum. *J. Med. Genet.*, **15**, 357.

Lynch, H. T., Frichot, B. C. & Lynch, J. F. (1978) Familial atypical multiple mole melanoma syndrome. *J. Med. Genet.*, **15**, 352.

Lynch, H. T., Frichot, B. C., Lynch, P. M., Lynch, J. & Guirgis, H. A. (1975) Family studies of malignant melanoma and associated cancer. *Surg. Gynecol. Obstet.*, **141**, 517.

Lynch, H. T., Fusaro, R. M., Pester, J. & Lynch, J. F. (1980) Familial atypical multiple mole melanoma (FAMMM) syndrome: Genetic heterogeneity and malignant melanoma. *Br. J. Cancer*, **42**, 58.

Lynch, H. T. & Fusaro, R. M. (1981) *Cancer-associated Genodermatoses.* New York: Van Nostrand Reinhold Co. (In press.)

Lynch, H. T. & Krush, A. J. (1968) Heredity and malignant melanoma: Implications for early cancer detection. *Can. Med. Assoc. J.*, **99**, 17.

Mulvihill, J. J. (1977) Genetic repertory of human neoplasia. In: *Genetics of Human Cancer* (Ed. Mulvihill *et al.*) New York: Raven Press. p. 517.

Norris, W. (1920) A case of fungoid disease. *Edinb. Med. Surg. J.*, **16**, 562.

Reimer, R. R., Clark, W. H., Greene, M. H., Ainsworth, A. M. & Fraumeni, H. F., Jr (1978) Precursor lesions in familial melanoma. *JAMA*, **239**, 744.

RODRIGUEZ-SAINS, R. S. (1980) Are concurrent or subsequent malignant melanomas in the skin and eye related or coincidental? *J. Dermatol. Surg. Oncol.*, **6**, 915.

TURKINGTON, R. W. (1965) Familial factors in malignant melanoma. *JAMA*, **192**, 77.

### ADDENDUM

Through the courtesy of Dr R. R. Hook, University of Missouri, Columbia, we have had an opportunity to evaluate malignant melanoma in the Sinclair miniature swine colony (Oxenhandler, R. W., Adelstein, E. H., Haigh, J. P., Hook, R. R., Jr. & Clark, W. H. (1979). Malignant melanoma in the Sinclair miniature swine: An autopsy study of 60 cases. *Am. J. Pathol.*, **96**, 707.) Interestingly, these animals have an excess of congenital malignant melanoma, multiple primary melanoma, multiple nevi, and a high rate of spontaneous regression of malignant melanoma. Preliminary evaluation of their premelanotic nevi by us showed histologic similarities to FAMMM moles. These animals may well provide a model for the study of the FAMMM syndrome.